# Investigation of carbohydrate-based molecules of *Theileria parva* parasites

**DOI:** 10.3389/fvets.2026.1816563

**Published:** 2026-06-08

**Authors:** Jeannine Kolakowski, Shi Yan, Johannes Stadlmann, Bernd Lepenies, Daniel Ngugi, Heather Harris, Dirk Werling

**Affiliations:** 1 Centre for Vaccinology and Regenerative Medicine, Royal Veterinary College, Hatfield, United Kingdom; 2 Institute of Parasitology, University of Veterinary Medicine Vienna, Vienna, Austria; 3 Institute of Biochemistry, University of Natural Resources and Life Sciences Vienna, Vienna, Austria; 4 Institute for Immunology, University of Veterinary Medicine Hannover, Hannover, Germany; 5 Department of Veterinary Sciences, Faculty of Veterinary Medicine, Ludwig-Maximilians-Universität München, Planegg, Germany

**Keywords:** Apicomplexa, East Coast fever (ECF), glycans and glycoconjugates, LC-MS/MS, Theileria parva

## Abstract

*Theileria parva* (Tp), the causative agent of East Coast Fever, poses a major threat to *Bos taurus* cattle in sub-Saharan Africa. Progress towards a stable, affordable, cross-protective subunit vaccine covering more than one major histocompatibility complex (MHC)-presented Tp antigen has been hindered by the limited knowledge of antigens. Given the importance of glycoconjugates in protozoan pathogen biology, especially during the invasion process, we investigated the glycosylation potential of Tp. Screening of Tp Muguga (TpM) schizonts with C-type lectin (-like) receptors showed that ruminant, but not murine, Macrophage C-type Lectin (MCL) recognises the parasite. Binding of ruminant MCL to TpM schizonts suggested the presence of carbohydrate-associated parasite surface molecules. Lectin staining further suggested the presence of terminal GlcNAc residues on the parasite surface which is in alignment with the identification of a minimal N-glycosylation machinery in TpM schizonts. LC-MS/MS analysis of HILIC HPLC and lectin-enriched peptides from parasite protein fractions yielded eight predicted N-glycopeptides and two predicted O-glycopeptides. Putative parasite O-glycans were assigned with high confidence, yet assignments of putative N-glycans to parasite proteins were less confident. Our data provide first insights into potential TpM glycoconjugates while confirmation of glycan composition and assignment requires additional analyses.

## Introduction

East Coast Fever (ECF) compromises bovine welfare and livestock productivity in sub-Saharan Africa significantly. High morbidity and mortality rates affect predominantly but not exclusively *Bos taurus* cattle breeds and pose a huge economic burden for especially small-holder pastoralists (1–3). The causative agent of ECF is the tick-transmitted apicomplexan parasite *Theileria parva* (Tp). Sporozoites of Tp are released into cattle hosts during blood meals of infected ticks from tick salivary glands and develop into schizonts after successful invasion of bovine lymphocytes. Schizonts transform and replicate together with their host cells which results in uncontrolled lymphocyte proliferation and migration (4). The exponential increase in the number of schizont-infected cells appears to stimulate a histiocytic inflammatory immune response in cattle that contributes to the main symptoms of ECF and often results in pulmonary oedema and respiratory failure due to severe vasculitis in affected animals (5, 6).

ECF could be a preventable disease. Current strategies involve frequent treatment of cattle with acaricides, resulting in a negative impact on the environment and food safety while promoting resistances in tick populations (7). The current “vaccine strategy,” the Infection and Treatment Method (ITM), involves inoculation of live Tp sporozoites in combination with long-acting oxytetracycline to prevent symptoms and death in treated animals (8, 9). The production process of ITM is laborious, time consuming and expensive, while the vaccine stabilates require a continuous cold chain and induce immune responses with limited cross-protectivity (10, 11). Several potential Tp antigens have been identified, and these are primarily denominated based on their molecular identification, function, and recognition by the bovine immune system. They are often labelled with a “Tp” prefix followed by a number or descriptive acronym, in addition to major sporozoite antigens such as PIM, p67 and p32. Immune protection from ECF is conferred by an MHC class I-restricted, cytotoxic CD8^+^ T cell response towards schizont-infected lymphocytes (12) that is induced and recalled by parasite-specific CD4^+^ T cells (13). Schizont-infected cells express both, MHC class I and II molecules on their surface which facilitates simultaneous presentation of parasite-derived CD8^+^ and CD4^+^ T cell antigens. However, protective CD8^+^ T cells from individual cattle only recognise a few dominant parasite antigens which can limit cross-protection from different Tp strains (14, 15). Screenings of a synthetic Tp peptide library identified that CD4^+^ T cells from individual animals recognise multiple parasite-derived antigens that include both conserved and polymorphic epitopes yet no parasite antigen was recognised by CD4^+^ T cells from all cattle tested (16).

The development of new treatment and prevention strategies for ECF requires a better understanding of the basic biology and biochemistry of the parasite. Glycoconjugates represent important virulence factors for many pathogens including protozoan parasites (17). They shield them from the environment, are involved in parasite–host interactions and contribute to the survival of parasites in their host cells. O-fucosylation of certain thrombospondin-repeat (TSR) domain containing proteins such as the thrombospondin-related anonymous protein (TRAP) and circumsporozoite protein (CSP) of *Plasmodium falciparum* (Pf) increases the invasion efficiency of hepatocytes by sporozoites (18). Asexual blood stages of Pf glycosylate proteins with truncated N-glycans (19) which is in alignment with their synthesis of sugar nucleotides including UDP-GlcNAc (20). Treatment of Pf trophozoites with tunicamycin, a nucleoside antibiotic that inhibits enzymes involved in the initial steps of N-glycosylation, induced delayed parasite death that could not be rescued with isoprenoid precursors and suggests that N-glycosylation is essential for this and potentially later Pf lifecycle stages (21). The identity and function of these N-glycoproteins, however, is still unclear. Other forms of glycosylation such as C-mannosylation that were reported in *Plasmodium* are reviewed in (22).

Even less is known about the glycobiology of Theileria parasites as they appear to possess very limited glycosylation capacity (19, 23, 24). The recent re-annotation of the Tp Muguga (TpM) genome and RNAseq analysis of TpM schizonts, however, identified a minimal N-glycosylation machinery that raises the questions whether and if so which molecules Tp parasites glycosylate (25). Glycopeptides are presented by MHC class I and II molecules and can be recognised specifically by T cells (26, 27). Glycosylation, however, can also affect MHC presentation and immune recognition of peptides by interfering with proteolytic processing of proteins (28) which makes it relevant for vaccine development.

In this study we aimed to investigate the glycosylation potential of TpM by screening parasites with glycan-binding proteins for glycosylated molecules that can support the development of a cross-protective subunit vaccine for ECF. Evidence that protective immunity to ECF is directed towards parasite-infected lymphocytes as well as limited accessibility of TpM sporozoites led us to focus on the schizont lifecycle stage of TpM. Our results thereby suggest the presence of parasite-derived N- and O-linked glycoproteins that require further confirmation in TpM and ultimately other Tp strains.

## Materials and methods

### Generation of a *T. parva* Muguga-infected cell line and cell culture

A *T. parva* Muguga (TpM) schizont-infected cell line was established by *in vitro* infection of bovine peripheral blood mononuclear cells (PBMCs, frozen from fresh) with TpM sporozoites from ground-up tick stabilates (GUTS, batch S71, 4 te mL^−1^). PBMCs were isolated from heparinised bovine whole blood (sampled under home office licence (PPL7009059)) as described in Holder et al. (29) and stored in liquid nitrogen for future use. GUTS were prepared for infection as described in Wilkie et al. (30). Schizont-infected cells were clonally expanded from the resulting group of infected cells, cultured in tissue culture medium (TCM, RPMI-1640 with GlutaMAX, 25 mM HEPES, 10% FBS, penicillin (100 U mL^−1^) / streptomycin (100 μg mL^−1^), 0.6 mM sodium pyruvate, 50 μM 2-mercaptoethanol) at 38°C and 5% CO_2_, and were split growth-dependently every 2–4 days. The presence of schizonts was confirmed regularly via Giemsa staining of culture smears.

### Bovine lectin array screening of isolated *T. parva* Muguga schizonts

TpM schizonts were enriched from 6 × 10^8^ infected cells as described by Wiens et al. (31) with slight modifications. Here, cells were incubated for 1 h at 38°C in the presence of 3 μg·mL^−1^ nocodazole to facilitate host cell membrane perforation. Perforated and mechanically lysed host cells were layered onto a Nycodenz step gradient consisting of 5 mL 5% Nycodenz solution underlaid with 5 mL of 30% and 3 mL of 40%. In contrast to Wiens et al., no calyculin A or DNase were used throughout this procedure.

Enriched schizonts were fixed with 4% PFA for 10 min at room temperature (RT) and were stained with SYBR Green Nucleic Acid stain (Invitrogen, 1:5,000 in 1 × PBS) for 30 min at RT. Stained parasites were resuspended in filtered 1 × PBS (0.22 μm syringe filter, Millex-GS). The lectin array screening was kindly performed by Prof Kurt Drickamer and Prof Maureen Taylor (Imperial College London) with their existing recombinant bovine lectin receptor carbohydrate recognition domains (CRDs) as described in Jegouzo et al. (32). Data were analysed using Microsoft Excel version 16.93.1 (Microsoft Corp) and GraphPad Prism version 10.4.0 (GraphPad Software, Inc). Values from duplicate wells were averaged and normalised to the maximum fluorescence signal measured.

### Flow cytometric CLR-hFc fusion protein screening

TpM schizonts were enriched from 5 × 10^8^ infected cells as stated above and fixed with 4% PFA for 10 min at RT. Fixed parasites were stained with 250 ng of recombinant C-type lectin receptor (CLR) human (h) Fc fusion proteins (Dectin-1, Dectin-2, MCL, MICL, Mincle, Langerin, DNGR, DCIR) (33, 34) in Lectin Binding Buffer (LBB, 50 mM HEPES, 5 mM MgCl_2_, 5 mM CaCl_2_, pH 7.4) for 1 h at RT. CLR-hFc-fusion proteins were detected with goat anti-hFc Alexa Fluor (AF) 680 (Jackson ImmunoResearch Europe Ltd., 1:300 in 1 × PBS) for 25 min at RT. Schizont DNA was stained with SYBR Green Nucleic Acid stain (Invitrogen, 1:17,000 in 1 × PBS) for 30 min at RT. Fully stained samples were resuspended in filtered (0.22 μm syringe filter, Millex-GS) sheath fluid (BD FACSFlow™ SheathFluid) and were analysed on a BD FACSAria™ Fusion cell sorter equipped with four lasers (355 nm, 405 nm, 488 nm, 640 nm) and standard emission filters. Control samples included unstained parasites and schizonts stained with SYBR Green Nucleic Acid stain alone. Binding of recombinant hFc without any CLR domain to parasites was considered background signal and was subtracted from the percentage of events bound by CLR-hFc fusion proteins. Data were analysed using the BD FACSDiva™ Software version 9.0, FlowJo™ Software version 10.8.1. and GraphPad Prism version 10.4.0. The gating strategy is shown in (Supplementary Figure 1A).

The detection of enriched TpM schizonts via flow cytometry was confirmed prior to this experiment via fluorescence activated cell sorting (FACS) and subsequent microscopy of sorted parasites. The schizont surface was stained with a mouse anti-polymorphic immunodominant molecule (PIM) primary antibody (IL-S40.3, 1:2,000 in 1 × PBS + 3% goat serum) and a goat anti-mouse IgG BV421 secondary antibody (Invitrogen, 1:300 in 1 × PBS + 3% goat serum). Parasite DNA was stained with SYBR Green nucleic acid stain (Invitrogen, 1:17,000 in 1 × PBS). Events with a fluorescence signal from both SYBR Green and anti-PIM antibody staining were sorted and visualised with the 100X oil lens of a Nikon Eclipse Ti2 fluorescence microscope (Supplementary Figure 2).

### Flow cytometric lectin screening

TpM schizonts were enriched from 1–1.5 × 10^8^ infected cells as described above and fixed with 4% PFA for 10 min at RT. Fixed parasites were incubated with WGA-647 (Invitrogen, 5 μg·mL^−1^) or biotinylated Concanavalin (Con) A (Bio-Rad, 5 μg·mL^−1^) in binding buffer (150 mM NaCl, 25 mM Tris–HCl, 0.1 mM CaCl_2_, pH 7.4) for 30 min at RT. Lectins either remained uninhibited or were preincubated with different concentrations of N-acetylglucosamine (GlcNAc; 2B Scientific, ligand for WGA) or *α*-methylmannoside (2B Scientific, ligand for ConA) in binding buffer for 1 h at RT. ConA was detected via incubation of samples with AF680-linked Streptavidin (Invitrogen, 1:400 in 1 × PBS) for 30 min at RT. Subsequently, parasites were stained with a mouse anti-PIM primary antibody (IL-S40.3, 1:2,000 in 1 × PBS + 1% goat serum) and a goat anti-mouse IgG BV421 secondary antibody (Invitrogen, 1:200 in 1 × PBS + 1% goat serum) for flow cytometric analysis. Samples were analysed on the BD FACSAria™ Fusion cell sorter, as mentioned above. Events with a fluorescent signal from PIM-based surface staining were assessed for lectin binding. Control samples included unstained parasites, schizonts stained with the secondary antibody, parasites stained with Streptavidin only, schizonts stained with either lectin alone and parasites stained with the secondary antibody and WGA-647 or Streptavidin. The gating (Supplementary Figure 1B) was performed using BD FACSDiva™ Software version 9.0 and FlowJo Software version 10.8.1. Data were analysed with GraphPad Prism version 10.4.0.

### Flow cytometric screening for O-GlcNAc

TpM schizonts were enriched from 1–1.5 × 10^8^ infected cells and analysed as for the flow cytometric lectin screening with modifications. Here, fixed parasites were stained with an anti-O-GlcNAc mAb (Invitrogen, clone RL2, 1:200 in 1 × PBS + 1% goat serum) and a goat anti-mouse IgG AF647 secondary antibody (Invitrogen, 1:300 in 1 × PBS + 1% goat serum) for 30 min at RT. Parasite DNA was stained with SYBR Green Nucleic Acid stain (Invitrogen, 1:17,000 in 1 × PBS) for 30 min at RT. Stained samples were analysed as stated above (Supplementary Figure 1C). Control samples included unstained parasites, schizonts stained with the secondary antibody alone as well as parasites stained with SYBR Green alone.

### TpM schizont whole parasite lysate

Whole protein was extracted from TpM schizonts (enriched from 2.29 × 10^9^ infected cells) and uninfected bovine lymphocytes. Lymphocytes were separated from PBMCs (2 × 10^8^, frozen from fresh) by letting the monocytes adhere to tissue culture plastic at 37 °C and 5% CO_2_ for 24 h. Parasites and cells were lysed in lysis buffer (150 mM NaCl, 10 mM Tris-HCl, 2% Triton X-114, 1 × Halt protease inhibitor cocktail, pH 7.4) for 1 h at 4 °C and were centrifuged at 8000×g for 10 min at 0 °C to pellet insoluble material (Pellet, kept separately). The supernatant was subject to three Triton X-114-based phase separations as described by Witschi et al. (35). All three aqueous phases (AP) were pooled before protein from both AP and detergent-rich phase (DP) was precipitated with five volumes of ice-cold methanol at −20 °C overnight. Precipitated protein was resuspended in molecular grade H_2_O for analysis via Western Blot and mass spectrometry (MS).

### Western blot analyses of TpM protein extract

For the detection of GlcNAc-containing glycoproteins, AP and DP samples were analysed by Western blot. De-N-glycosylation of AP and DP samples was performed by overnight incubation of 80 μg of proteins with 3 units of PNGase F (Roche) at 37 °C. Samples without PNGase F treatment were incubated at 37 °C under the same conditions as the treated samples. Ribonuclease (RNase) B from bovine pancreas (Sigma-Aldrich) was employed as a positive control for de-N-glycosylation. Additionally, RNase B was remodelled by endoglycosidase (Endo) H (Roche) at 37 °C overnight to generate a truncated N-glycoform (GlcNAcβ-Asn) and was used to confirm lectin binding specificity. BSA conjugated to a synthetic GlcNAcβ2Man-R disaccharide served as another control (36).

Briefly, samples were denatured in SDS-loading buffer containing 0.1 M DTT at 95 °C for 10 min and run on 12.5% SDS-PAGE gels. Proteins were transferred onto a nitrocellulose (NC) membrane for 7 min at 25 V using a semi-dry blotting system (Bio-Rad). Transfer of proteins to the NC membrane was confirmed via ponceau S (Sigma-Aldrich) staining (Supplementary Figure 3A). Post blocking with 0.5% BSA in TBST buffer (0.1 M NaCl, 0.1 M Tris-HCl, 0.05% Tween-20, pH 7.4) for 1 h at RT, the blot was incubated for 1 h at RT with a biotinylated wheat germ agglutinin (WGA, 10 μg·mL^−1^, Vector Laboratories) in 1 × PBS supplemented with 2 mM CaCl_2_, and subsequently incubated with an alkaline phosphatase-conjugated anti-biotin secondary antibody (1,10,000, Sigma-Aldrich). SIGMAFAST™ BCIP®/NBT was used as the substrate for colour development.

For the detection of O-GlcNAcylated proteins, AP and DP samples were heated in LDS sample buffer (NuPAGE Novex, Invitrogen) containing 0.1 M DTT for 10 min at 70 °C and were run on 4–12% Bis Tris gels in 1 × MOPS buffer (NuPAGE Novex, Invitrogen). Protein was transferred onto NC membranes (Amersham Protran, 0.2 μm) at 10 V overnight. Loading of protein was confirmed via Ponceau S staining (Supplementary Figures 3B,C). Membranes were blocked in TBST + 3% BSA for 1 h at RT and were incubated with anti-O-GlcNAc mAb (Invitrogen, clone RL2, 1:1,000 in TBST + 3% BSA) for 5 h at RT and goat anti-mouse IgG HRP secondary antibody (Bio-Rad, 1:2,500 in TBST + 3% BSA) for 1 h at RT. Ponceau and antibody stainings were visualised on an iBright1500 (Invitrogen). Control samples included 0.5 μg of recombinant human Tau-441 expressed in HEK293 cells (Sigma-Aldrich) and 0.5 μg of BSA (Pierce Protein Standard).

### Mass spectrometry of glycopeptides from TpM protein extract

To determine potential parasite glycans, proteins from AP and DP fractions of TpM schizonts were subjected to glycoproteomic analyses using mass spectrometry (MS). Briefly, protein samples were denatured in reducing buffer (8 M urea, 50 mM ammonium bicarbonate, 5 mM DTT, pH 7.8) and treated with trypsin (Promega) overnight at 37 °C. The resulting peptides were purified using hand-packed C18 cartridges (50% acetonitrile with 0.1% trifluoroacetic acid as eluent) and evaporated to dryness using a speed-vac concentrator. Dried peptides were resuspended in HPLC-grade water and analysed on an Autoflex Speed MALDI-TOF MS (Bruker Daltonics) using α-cyano-4-hydroxycinnamic acid or 6-aza-2-thiothymine as matrix. The instrument was calibrated with the Bruker Peptide Calibration Standard II.

Additionally, AP and DP samples were analysed by nanoLC-ESI-MS (/MS), using a Q Exactive HF Orbitrap mass spectrometer (Thermo Fisher Scientific) coupled to a nano-HPLC Ultimate 3,000 RSLC system (Dionex) for peptide separation. MS full scans were performed in the ultrahigh-field Orbitrap mass analyser in ranges *m*/*z* 350–2,000 with a resolution of 60,000, the maximum injection time (MIT) was 50 ms and the automatic gain control (AGC) was set to 3*e*^6^. The top ten intense ions of each MS scan were selected for gas-phase fragmentation using high energy collision dissociation (HCD). For each MS/MS scan, the AGC was set at 5*e*^4^ and the MIT was 50 ms. Dynamic exclusion of precursor ion masses over a time window of 30s was used to reduce MS/MS data redundancy.

Overall glycopeptide populations of AP and DP samples were enriched using hydrophilic interaction liquid chromatography (HILIC), as described recently (37). In brief, dried (glyco)peptides were re-suspended in 50 μL of 80% acetonitrile containing 1% trifluoroacetic acid (TFA) and fractionated using a TSK-Amide 80 column (4.6 × 250 mm, 5 μm, Tosoh). A linear gradient was developed from 80% acetonitrile with 0.1% TFA to 50% acetonitrile with 0.1% TFA over 30 min, at a flow rate of 1 mL/min. Fractions of 1 mL were collected using a fraction collector (Shimadzu). Late-eluting HILIC fractions (minutes 20–39) were pooled and lyophilised prior to LC–MS/MS analysis.

Wheat germ agglutinin (WGA) binding glycopeptide ligands were enriched from C18-purified (glyco)peptide fractions (DP and Pellet) by lectin affinity chromatography (LAC), using a WGA-based glycoprotein isolation kit (Thermo Fisher Scientific), following the manufacturer’s instructions. LAC eluted fractions were desalted using C18 cartridges as stated above and lyophilised.

Enriched (glyco)peptides were resuspended in 20 μL of 0.1% formic acid and separated by reversed-phase nano-LC using a nanoEase M/Z HSS T3 column (100 Å, 1.8 μm, 300 μm × 150 mm, Waters). MS/MS analysis was performed on an Orbitrap Exploris 480 mass spectrometer (Thermo Fisher Scientific) equipped with a standard H-ESI source, operated in positive ion mode. MS spectra were acquired in the *m*/*z* range of 350–1,500 at a resolution of 60,000. Data-dependent MS/MS acquisition was triggered for precursor ions with charge states 2–6 and intensities above 800,000, using an isolation window of 1.4 *m*/*z* (offset 0.5 *m*/*z*), with a normalised AGC target of 200%. Fragmentation was performed using stepped HCD. MS/MS scans were acquired at a resolution of 30,000 with the first fixed mass set at *m*/*z* 120.

Raw data of C18-purified AP and DP samples were searched against proteomes of *T. parva* (Taxon ID: 5875; 2,790 entries) and *B. taurus* (Taxon ID: 9913; 37,504 entries) using Proteome Discoverer (version 2.4.1.15; Thermo Fisher Scientific). Furthermore, the raw data were analysed using the FragPipe software suite (v22.0), incorporating MSFragger (v4.1) and Philosopher (v5.1.1). N- and O-glycopeptides were identified using the glyco-N-HCD and glyco-O-HCD workflows with default parameters, allowing one missed tryptic cleavage. Cysteine carbamidomethylation was set as a fixed modification, while methionine oxidation and HexNAc (including neutral loss) were specified as variable modifications. False discovery rates (FDR) were controlled at 1% at the PSM, peptide, and protein levels. Glycan searches were performed using Byonic glycan databases (309 N-glycan and 78 O-glycan compositions), and glycan assignments were filtered at a 5% FDR. PTM-Shepherd was used in “Glyco Search” mode, and only glycosylation sites with a localisation probability >0.75 were considered confident.

Prediction of signal peptides and transmembrane domains as well as N-glycosylation sites for identified proteins was performed with the SignalP-6.0 (38), Deep TMHMM-1.0 algorithms (39) and NetNGlyc-1.0 (40), respectively, developed by DTU Health Tech. The mass spectrometry proteomics data have been deposited to the ProteomeXchange Consortium via the PRIDE partner repository with the dataset identifier PXD070746.

## Results

### Surface molecules of TpM schizonts are recognised by recombinant ruminant MCL

**Figure 1:**
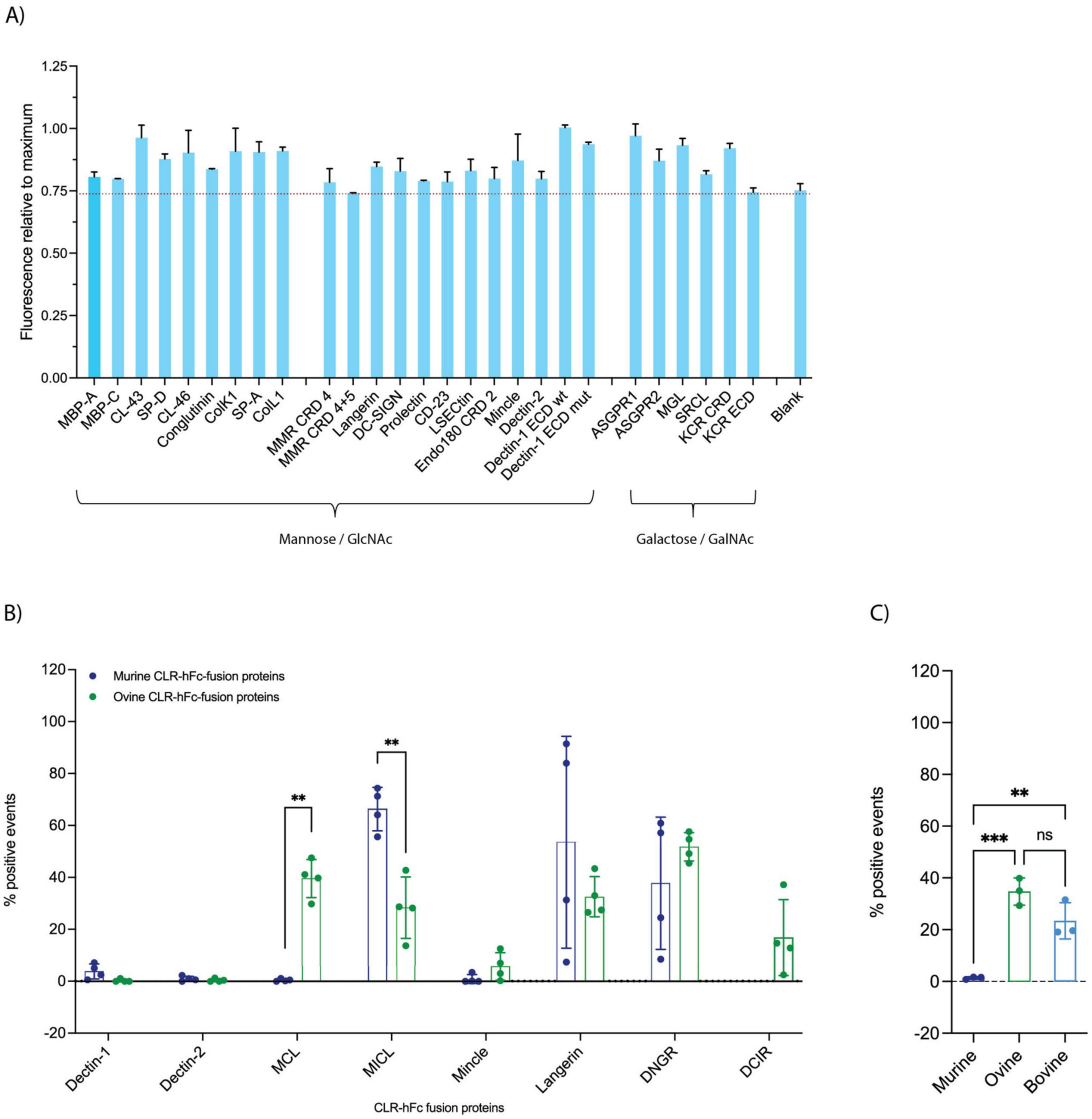
Screening of enriched *TpM* schizonts with mammalian lectins. **(A)** ELISA-based screening of SYBR Green-stained *TpM* schizonts with a bovine lectin array. Parasites were enriched from 6 × 10^8^ infected cells for this screening. The results are shown relative to the maximum fluorescence units for binding to the wild-type (WT) extracellular domain of Dectin-1. The mean for duplicate samples is represented by bars and the standard deviation (SD) by error bars. The red dotted line represents the level of background signal. Flow cytometric screening of *TpM* schizonts with **(B)** different CLR-hFc-fusion proteins and **(C)** MCL-hFc-fusion protein from three different species. Parasites were enriched from 5 × 10^8^ infected cells for these experiments. The percentage of bound parasites for each of four/three experiments is shown as filled circles. The mean is represented by bars and the SD by error bars. Data were analysed using a **(B)** two-way ANOVA with Geisser–Greenhouse correction and Šídák multiple comparisons test (**p < 0.003) or a **(C)** one-way ANOVA with Tukey multiple comparisons test (ns, not significant, **p = 0.0043, ***p = 0.0005).

We first screened *Theileria parva* Muguga (TpM) schizonts that had been enriched from their respective host cells with an array of recombinant C-type carbohydrate recognition domains (CRDs) from 23 different bovine lectin receptors to confirm the presence of glycosylated surface molecules. Array members had been selected based on their immunological relevance for cattle and included receptor CRDs with a mannose- or galactose-type ligand binding site to cover a broad glycan spectrum. While binding of fluorescently labelled parasites was detected to 25 out of the 27 proteins in the array (Figure 1A), the fluorescent signal from bound schizonts was less than two-fold higher than the background signal (the background signal in Figure 1A is indicated by the red dotted line). The rather small increase in signal intensity from bound parasites compared to the background signal stood in contrast to previous results from bacterial samples that had been screened with the same receptor array and suggested weak or unspecific interactions of recombinant CRDs with the parasites (32). We therefore analysed enriched TpM schizonts with two libraries of recombinant extracellular domains of C-type lectin (-like) receptors fused to the Fc region of human IgG1 (CLR-hFc-fusion proteins) in a flow cytometric assay (34). These libraries added immunologically relevant lectins from other species that could possess a different ligand spectrum and allowed us to increase the sensitivity of our analysis to account for low glycosylation levels of parasite surface molecules. The flow cytometric screening confirmed some of our initial results including very weak or no binding of macrophage inducible C-type lectin (Mincle), Dectin-1 and Dectin-2 to the parasites, respectively (Figure 1B). Other CLR-hFc-fusion proteins such as those with the extracellular domains of Langerin and the dendritic cell immunoreceptor (DCIR) recognised the schizonts rather inconsistently. In contrast, ovine but not murine macrophage C-type lectin (MCL) bound to enriched parasites (adjusted *p*-value of 0.0019) while murine Myeloid inhibitory C-type lectin-like receptor (MICL) bound to a significantly higher percentage of schizonts than ovine MICL (adjusted *p*-value of 0.0027) (Figure 1B). Recognition of schizonts by bovine MCL (the bovine CLR-hFc-fusion protein library is described in (41)) was confirmed subsequently via flow cytometry as this receptor was not represented on the bovine lectin array (Figure 1C). MICL was reported to recognise crystalline ligands such as monosodium urate (42) and hemozoin (43) as well as mycobacterial mycolic acids (44) which led us to focus on MCL. Interactions of ovine and bovine MCL with enriched TpM schizonts suggested the presence of carbohydrate-associated molecules on the surface of the parasites and were in alignment with the host specificity of TpM for ruminants.

### Lectin screening of TpM schizonts suggests the presence of N-glycoproteins

**Figure 2:**
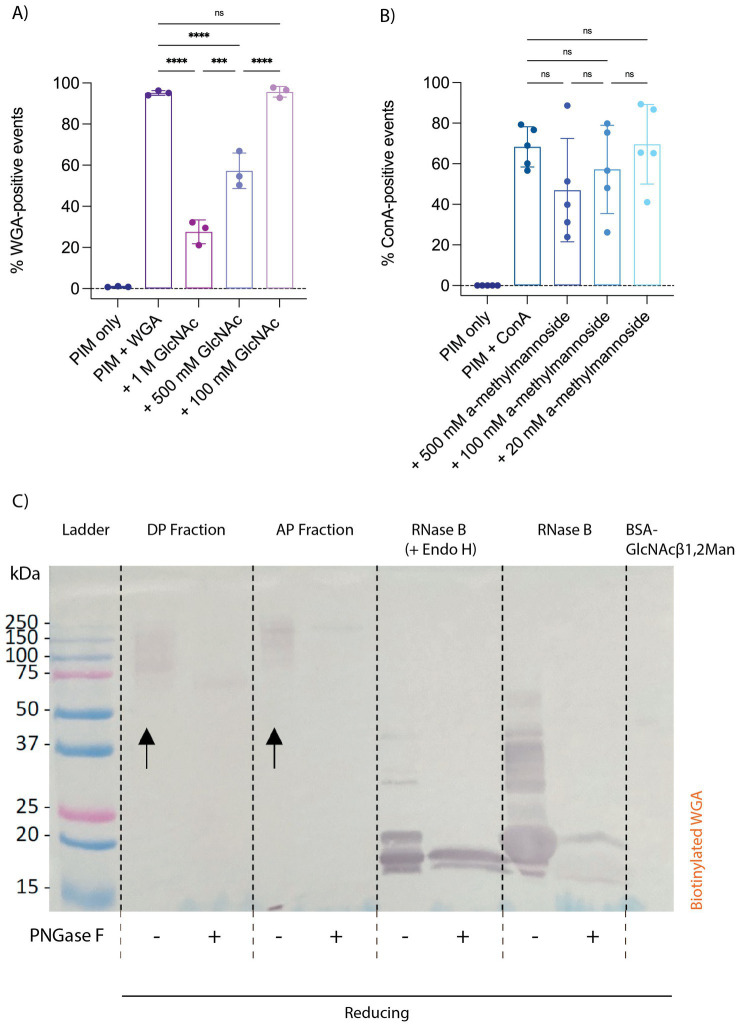
Screening of enriched *TpM* schizonts for N-glycans. Flow cytometric screening of *TpM* schizonts with **(A)** WGA and **(B)** ConA. Parasites were enriched from 1–1.5 × 10^8^ infected cells for these screenings. Events with a fluorescent signal from staining of an abundant parasite surface protein (PIM) were analysed for lectin binding. The results are shown relative to the percentage of PIM-stained events. The percentage of lectin-stained parasites for each of three/five independent experiments is shown as filled circles. The mean is represented by bars and the SD by error bars. Data were analysed using a one-way ANOVA with Tukey multiple comparisons test (ns, not significant, ***p < 0.0005, ****p < 0.0001) and a Brown-Forsythe and Welch ANOVA with Dunnett multiple comparisons test (ns = not significant), respectively. **(C)** Western blot analysis of DP (detergent-rich phase) and AP (aqueous phase) fractions of *TpM* schizont whole parasite lysate. DP and AP samples were either untreated (−) or de-N-glycosylated with PNGase F (+) to confirm WGA binding specificity. Bovine ribonuclease (RNase) B, the N-glycosylated form of bovine pancreatic RNase, was used to confirm de-N-glycosylation by PNGase F. Treatment of RNase B with endoglycosidase (Endo) H generated a truncated N-glycoform that was used to confirm WGA binding specificity further. BSA-GlcNAcβ1,2Man was included as negative control in line with (36).

The current knowledge of the ligand spectrum of ruminant MCL is limited and demanded a targeted search for specific residues of potential parasite surface glycans. MCL is a type-II transmembrane receptor and a member of the Dectin-2 family (45). Human MCL has the capacity to bind GlcNAc residues next to mannose and fucose residues (46). GlcNAc residues are cornerstones of the eukaryotic N-glycosylation pathway, three enzymes of which were identified in the most recent RNAseq analysis of TpM schizonts (25). We therefore screened the surface of enriched parasites with wheat germ agglutinin (WGA), a plant lectin that binds specifically to GlcNAc and sialic acid residues (47, 48). Flow cytometric analysis of enriched TpM schizonts after incubation with WGA showed binding of the lectin to the parasites (Figure 2A). Treatment of WGA with different concentrations of GlcNAc prior to its incubation with the schizonts reduced the percentage of bound parasites in a dose-dependent manner and indicated specific recognition of GlcNAc residues by WGA. In contrast, binding of concanavalin A (ConA), a plant lectin with a preference for mannose and glucose residues (49), to enriched parasites could not be inhibited consistently via incubation with different concentrations of one of its ligands, α-methylmannoside (Figure 2B). This suggested that ConA did not interact with mannose residues.

Tretina et al. (25) identified orthologues of three glycosyltransferases (ALG7, ALG13, ALG14) in their annotation of the TpM genome and the corresponding transcripts in their RNAseq analysis of TpM schizonts. In other eukaryotes, these enzymes add the initial two GlcNAc residues of N-glycans onto the lipid carrier molecule dolichol phosphate (Dol-P) in the membrane of the endoplasmic reticulum where the lipid-linked oligosaccharide (LLO) precursor can be extended and ultimately transferred onto the asparagine residue of a protein by an oligosaccharyltransferase (OST). However, an ortholog of an OST such as the STT3 of the oligosaccharyltransferase complex in *P. falciparum* could not be detected in the TpM genome annotation. We therefore analysed protein extracts from enriched TpM schizonts to assess whether parasite molecules recognised by WGA could be N-glycoproteins.

To expand the repertoire of analysed proteins, in particular membrane-bound proteins, we subjected whole parasite lysate from enriched TpM schizonts to Triton X-114-based phase separations and analysed fractions enriched for either hydrophobic (DP, detergent-rich phase) or hydrophilic (AP, aqueous phase) proteins separately. Proteins in both fractions, ranging between 70 and 250 kDa, were recognised by WGA (Figure 2C, black arrows). This signal was nearly abolished for samples that had been de-N-glycosylated via PNGase F treatment prior to WB analysis and confirmed specific recognition of N-linked glycans by the lectin (Figure 2C). These results indicated that ligands bound by WGA could be TpM schizont-derived N-glycoproteins.

### Antibody screening of TpM schizonts suggests the presence of O-GlcNAcylated proteins

**Figure 3:**
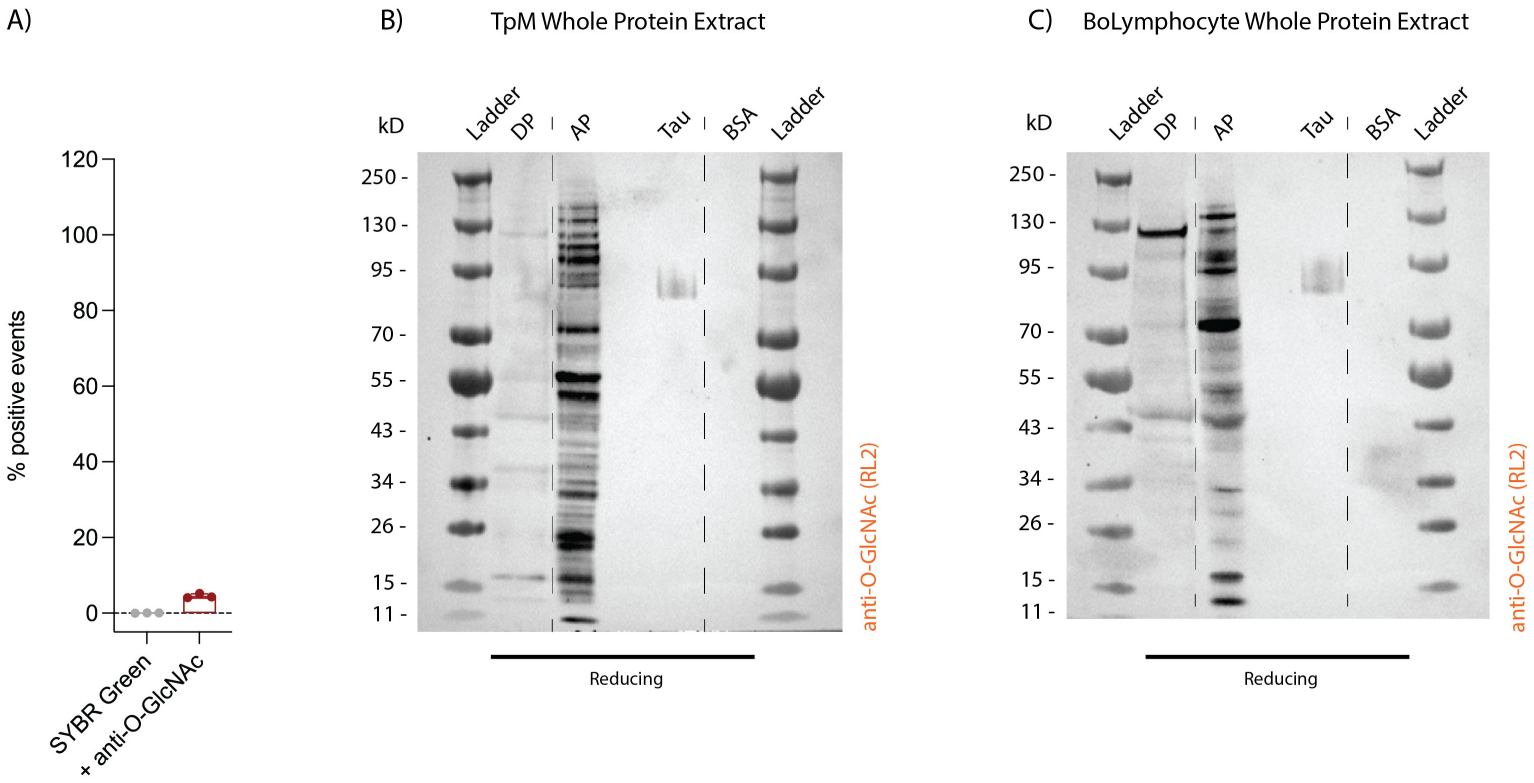
Screening of enriched TpM schizonts for O-GlcNAc. **(A)** Flow cytometric screening of enriched TpM schizonts for O-GlcNAcylated surface molecules. Events with a fluorescent signal from SYBR Green staining were analysed for antibody binding. The results are shown relative to the percentage of SYBR Green stained events. The percentage of stained parasites for each of three independent experiments is shown as filled circle. The mean is represented by bars and the SD by error bars. Western blot analysis of DP (detergent-rich phase) and AP (aqueous phase) protein fractions from **(B)** enriched TpM schizonts and **(C)** uninfected bovine lymphocytes with an anti-O-GlcNAc monoclonal Ab (clone RL2). Recombinant human Tau-441 expressed in HEK293 cells served as positive control, BSA was included as negative control.

A much more transient and dynamic form of glycosylation than N-glycosylation is the β-linkage of individual GlcNAc residues to serine or threonine residues of proteins via O-GlcNAc transferases (OGTs). WGA can recognise both N- and O-linked glycans carrying terminal GlcNAc, yet O-GlcNAcylation occurs mostly as posttranslational modification of intracellular proteins including certain proteins from *Toxoplasma gondii* and Pf (50, 51). The results of a flow cytometric screening of enriched TpM schizonts for O-GlcNAcylated surface molecules were in agreement with this observation as only 4% to 5% of parasites were recognised by the O-GlcNAc-specific monoclonal antibody (Figure 3A). However, the same antibody recognised many proteins especially in the AP fraction of schizont-derived lysate (Figure 3B). The corresponding WB analysis of AP and DP protein fractions from uninfected bovine lymphocytes resulted in a different staining pattern compared to parasite-derived samples, thereby suggesting that Tp schizonts could possess intracellular proteins that are O-GlcNAcylated (Figure 3C).

### LC-MS/MS analysis of TpM protein fractions identified primarily host-associated N- and O-glycosylated proteins

**Figure 4:**
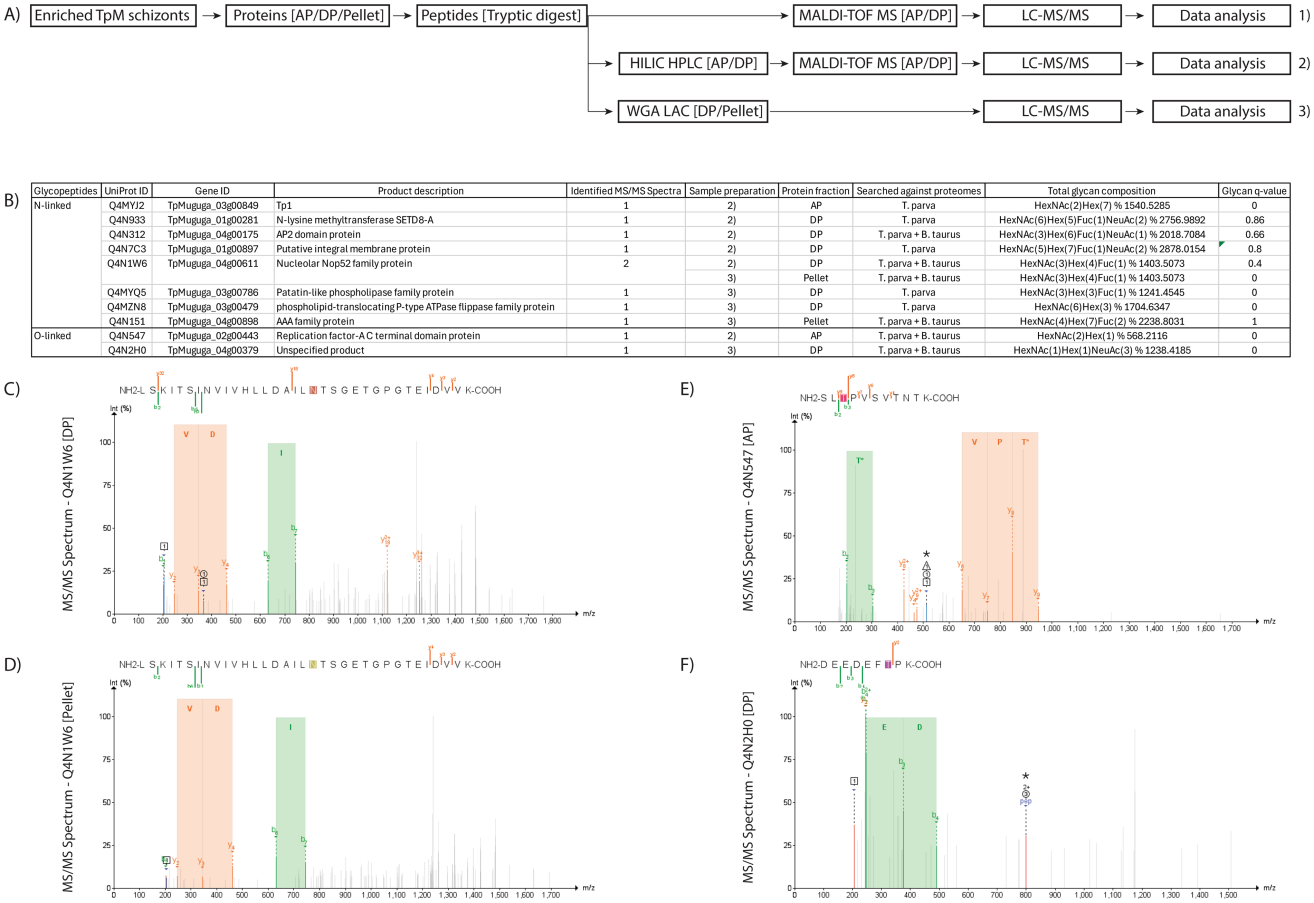
Mass spectrometric analysis of TpM schizont protein fractions. **(A)** Sample preparation and analysis strategies used to identify TpM schizont-derived glycoproteins and their glycan composition. **(B)** Parasite-specific N- and O-linked glycopeptides identified in schizont-derived protein fractions via LC–MS/MS and the FragPipe software version 220. The numbers in the column “Sample preparation” refer to those on the right side of the strategies shown in **(A)**. Examples of parasite-specific MS/MS spectra with putative **(C,D)** N-glycan or **(E,F)** O-glycan modifications. Fragment ion annotations were generated using FragPipe-PDV. Detected b-ions (green) and y-ions (orange) are highlighted. Glycan fragment ions are indicated using standard symbolic nomenclature (square, HexNAc; circle, hexose; triangle, fucose). Asterisks (*) denote non-informative or pseudo-glycan fragment annotations automatically assigned by the software and not consistent with the inferred glycan composition.

To identify glycosylated proteins and their glycan composition further, protein fractions from TpM schizonts were analysed by MS. AP and DP fractions from four separate parasite enrichments (from a total of 2.29 × 10^9^ infected cells) and protein extractions were combined for this analysis. Peptides were generated via tryptic digest, purified with C18 cartridges and subject to (1) MALDI-TOF MS followed by LC-MS/MS to identify proteins (Supplementary Table 1) and (2) glycopeptide enrichment followed by MS analyses. Glycopeptide enrichments were done via either HILIC-HPLC (Supplementary Figure 4) or WGA-based lectin affinity chromatography (LAC) for subsequent LC-MS/MS. Sample preparation and analysis strategies are shown in (Figure 4A). Raw LC-MS/MS data were searched against *T. parva* and *Bos taurus* proteomes to verify that such glycoproteins are parasite- rather than bovine host cell-derived. Search results were summarised in (Supplementary Table 2).

Only a small number of glycopeptides (fewer than 60) were identified to carry N-glycans. Most of the PSM hits were thereby associated with the DP fraction. As judged by the predicted glycan compositions, the asparagine residues of N-glycosites carried mostly oligomannosidic glycans (Hex_5-9_HexNAc_2_). Fucosylated and sialylated complex-type N-glycans were also identified for a few spectra. Most identified glycopeptides were associated with bovine proteins, some of which are known to be N-glycosylated including endoplasmin (Q95M18) and the cation-dependent mannose-6-phosphate receptor (P11456). In total, nine MS/MS spectra, corresponding to eight different proteins, were identified to be parasite-specific, carrying various types of putative N-glycan modifications including fucose and sialic acid (Figures 4B–F). The anticipated truncated forms of N-glycan modification (HexNAc_1-2_) were not detected in *T. parva* samples. O-glycopeptides were identified with an even smaller number of PSM than N-glycopeptides, the majority of which belonged to bovine proteins such as endoplasmic reticulum resident protein 44 (Q3T0L2) and the nucleus-localised histone H2B (F1MJU1). Only two MS/MS spectra were identified as parasite-derived O-glycoproteins (Q4N547 and Q4N2H0) (Figures 4E,F), carrying presumably mucin type O-glycans (Figure 4B) as judged by the observed glycan compositions.

## Discussion

Glycosylation is an important and abundant modification of proteins and lipids in eukaryotes, including apicomplexan parasites. It impacts the processing, stability and function of modified molecules that in turn facilitate the establishment of parasites in their respective hosts (17, 52, 53). The impact of glycosylation on antigen presentation and recognition by the innate and adaptive immune systems of host organisms further highlights its importance for vaccine development (26–28). The aim of this study was to expand our limited knowledge of the glycosylation potential of TpM to identify potential glycoproteins that are suitable to support the development of a cross-protective subunit vaccine for ECF.

To do so, we first assessed whether specific bovine carbohydrate-recognition receptors interact with Tp parasites and identified ruminant MCL as a receptor that binds enriched TpM schizonts. This suggested the presence of carbohydrate-associated parasite surface molecules (Figure 1). CRDs of murine and ruminant MCL show sequence and structural differences in their ligand binding sites as predicted by AlphaFold3 Server (54) (Supplementary Figure 5) which could explain the binding of enriched TpM schizonts by ruminant but not murine MCL. Screenings of enriched TpM schizonts with WGA and ConA further indicated that potential surface glycans could contain GlcNAc, but not mannose residues (Figure 2). This was in alignment with the identification of a minimal N-glycosylation machinery that has the potential to generate glycolipids with one or two GlcNAc residues (25) while gene sequences for the ALG enzymes involved in the assembly of mannosylated LLO appear to be absent from the schizonts. The absence of detectable OST gene homologues raised the question if TpM schizonts can generate N-glycoproteins or if MCL recognition is associated with other surface glycoconjugates, such as glycolipids.

Similar to glycoproteins, glycolipids can be recognised specifically by lectin receptors including MCL, MICL and Mincle. All three receptors were reported to bind mycobacterial lipids such as TDM (cord factor) amongst others (44, 55, 56) and might be involved in the recognition of other glycolipids. In contrast to ruminant MCL, recombinant Mincle from either of the three species tested did not recognise enriched TpM schizonts in this study (Figure 1). In addition, MICL lacks the conserved amino acids that form a Ca^2+^ binding site in its CRD. This group of receptors recognises diverse endogenous and exogenous ligands that may or may not contain carbohydrates in a Ca^2+^-independent manner (57). This study did not investigate MICL binding further due to its broad range of potential ligands which also includes crystalline molecules. However, binding of enriched TpM schizonts by MICL might still warrant further investigations, especially during explorations of the parasite glycolipidome. Specific recognition of glycolipids by NKT cells via presentation on CD1d molecules of antigen presenting cells (58) underlines how a better understanding of the putative TpM glycolipidome can support the development of a cross-protective vaccine for ECF.

Despite the incomplete set of genes required for protein N-glycosylation in TpM, analyses of proteins in schizont lysate shed light on the presence of N-glycoproteins and O-GlcNAcylated proteins in parasite-derived samples (Figures 2, 3). However, the enrichment method of TpM schizonts from infected bovine lymphocytes is vulnerable to contamination of samples with host-derived glycoconjugates. This was confirmed by LC–MS/MS analysis of AP and DP protein fractions from TpM schizont lysates that identified parasite- and host cell-derived peptides in both fractions (Supplementary Table 3). We therefore analysed putative glycopeptides in protein fractions of parasite lysate after tryptic digest rather than released glycans as it was done previously for *Plasmodium* parasites (19). LC–MS/MS analysis of either fraction post HILIC-HPLC and WGA enrichment further highlighted glycopeptides from both bovine host cells and TpM schizonts (Supplementary Table 2). Eight schizont-derived peptides were predicted to be N-glycosylated while two were predicted to be O-glycosylated by FragPipe V.220 (Figure 4B, Supplementary Table 4). The unexpected diversity of glycan compositions, the relatively high q-values indicating lower confidence in glycan assignment (Figure 4B), and the predicted absence of signal peptides and transmembrane domains from most putative N-glycoproteins as well as all putative O-glycoproteins despite the presence of multiple N-glycosites on each protein (Supplementary Table 4), underlined the need for additional investigations. These would require more parasite material and efficient glycoprotein enrichment steps prior to LC-MS/MS analysis. The non-template driven nature of glycan biosynthesis and the current inability to edit the genome of TpM schizonts makes it more difficult to analyse the glycoproteome of TpM schizonts independently from host cell glycoproteins (59). In contrast to the N-glycoproteins, glycans of one putative O-glycoprotein of TpM schizonts were assigned with a low FDR. This included one HexNAc, one Hex and three NeuNAc residues across all glycosylation sites. While O-glycosylation including O-GlcNAcylation can occur outside of the conventional secretory pathway, this finding and the unexpected presence of sialic acid residues has to be investigated with additional analyses as well.

## Data Availability

The datasets presented in this study can be found in online repositories. The names of the repository/repositories and accession number(s) can be found in the article/Supplementary material.

## Supplementary material

SUPPLEMENTARY FIGURE 1 Gating strategies for flow cytometric screenings of enriched TpM schizonts for glycosylated surface molecules. TpM schizonts were enriched from infected cells and stained with (A) different CLR-hFc-fusion proteins, (B) WGA with or without prior incubation with different concentrations of GlcNAc or (C) an anti-O-GlcNAc-specific mAb. Flow cytometric identification of all events compared to filtered sheath fluid alone was followed by two doublet discriminations (gating strategy). Single events with a fluorescent signal from (A + C) DNA staining or (B) anti-PIM staining were assessed for either lectin or mAb binding.

SUPPLEMENTARY FIGURE 2 Detection of enriched TpM schizonts via flow cytometry. TpM schizonts enriched from 1 × 10^8^ infected cells were stained for the abundant polymorphic immunodominant molecule (PIM) on the parasite surface and schizont DNA. (A) Identification of all events in comparison to filtered sheath fluid alone on a BD FACSAria^TM^ Fusion cell sorter was followed by two doubled discriminations (gating strategy). Events with a fluorescent signal from both PIM and DNA staining (Q2) were sorted for analysis via fluorescence microscopy. The gating for such events was performed conservatively to minimise the analysis and sorting of potential host cell contaminants. Control samples included unstained parasites (red) and schizonts stained with DNA stain and secondary antibody alone (blue). (B) Microscopic analysis of sorted events with the 100X oil lens of a Nikon Ti2 microscope showed round areas of about 5 μm with several green fluorescing foci from SYBR Green staining and a violet surface fluorescence from anti-PIM staining.

SUPPLEMENTARY FIGURE 3 Loading controls for Western Blot analyses of protein extract. (A) Ponceau staining of the Western Blot membrane shown in Figure 2C confirmed approximately equal loading of untreated and de-N-glycosylated samples for subsequent probing of the membrane with WGA. Bovine ribonuclease (RNase) B with an unmodified (no Endo H treatment) or truncated N-glycoform (post Endo H treatment) was used to confirm de-N-glycosylation of samples via PNGase F and WGA binding specificity, respectively. BSA-GlcNAcïβ¢1,2Man served as negative control. Ponceau staining of the Western Blot membranes shown in Figures 3B,C were imaged in grey scale on an iBright1500 and confirmed approximately equal loading of DP and AP protein fractions from (B) enriched TpM schizonts and (C) uninfected bovine lymphocytes. Recombinant human Tau-441 and BSA served as positive and negative control, respectively. Tau-441 ran between 80 and 100 kDa due to its glycosylation which led to its very faint ponceau staining.

SUPPLEMENTARY FIGURE 4 HILIC-HPLC and MALDI-TOF MS analysis of TpM schizont peptide fractions for subsequent LC-MS/MS. (A) HILIC-HPLC chromatograms of TpM schizont-derived AP and DP tryptic peptides as well as of control samples RNase B and endoglycosidase (Endo) H-treated RNase B. (B) MALDI-TOF MS spectra of glycopeptides from the HPLC-enriched TpM schizont DP fraction, displaying peptides carrying hexose oligomers (*χ*= 162). Red and blue arrows indicate two peptide series.

SUPPLEMENTARY FIGURE 5 Comparison of the ligand binding site of the CRD of bovine, ovine and murine MCL.3D protein structures of the CRD from bovine, ovine (AA82-213) and murine (AA83-218) MCL were generated with AlphaFold3 Server and assessed using ChimeraX-1.9. The region that would contain the canonical sugar-binding site of C-type carbohydrate-recognition domains of other C-type lectin receptors (red rectangle) was identified as described in (32).

SUPPLEMENTARY TABLE 1 Protein identification.

SUPPLEMENTARY TABLE 2 Summary of FragPipe search results.

SUPPLEMENTARY TABLE 3 Numbers of parasite- and host cell-derived proteins identified in AP and DP protein fractions via LC-MS/MS.

SUPPLEMENTARY TABLE 4 Predicted number of glycosites, signal peptides, and transmembrane domains (TMDs) for putative parasite-derived glycopeptides.
