# Supplementary material for: Investigation of carbohydrate-based molecules of Theileria parva parasites
Source: Front Vet Sci. 2026 Jun 8;13:1816563. doi: 10.3389/fvets.2026.1816563 (PMC13283990; doi:10.3389/fvets.2026.1816563)
Supplement: SUPPLEMENTARY FIGURE 1 — Gating strategies for flow cytometric screenings of enriched TpM schizonts for glycosylated surface molecules. TpM schizonts were enriched from infected cells and stained with (A) different CLR-hFc-fusion proteins, (B) WGA with or without prior incubation with different concentrations of GlcNAc or (C) an anti-O-GlcNAc-specific mAb. Flow cytometric identification of all events compared to filtered sheath fluid alone was followed by two doublet discriminations (gating strategy). Single events with a fluorescent signal from (A + C) DNA staining or (B) anti-PIM staining were assessed for either lectin or mAb binding. [file Supplementary_file_1.zip › Supplementary_Material/Supplementary Figures.pdf]

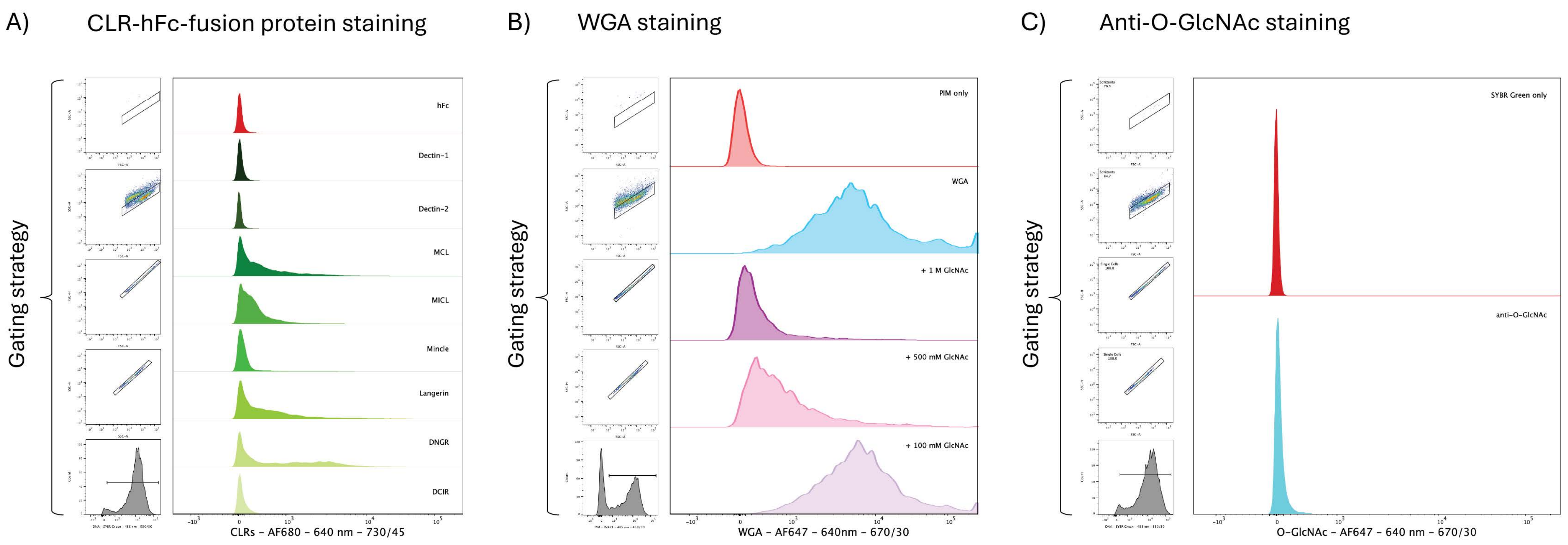

Supplementary Figure 1 Gating strategies for flow cytometric screenings of enriched TpM schizonts for glycosylated surface molecules
TpM schizonts were enriched from infected cells and stained with A) different CLR-hFc-fusion proteins, B) WGA with or without prior incubation with different concentrations of GlcNAc or C) an anti-O-GlcNAc-specific mAb. Flow cytometric identification of all events compared to filtered sheath fluid alone was followed by two doublet discriminations (gating strategy). Single events with a fluorescent signal from A + C) DNA staining or B) anti-PIM staining were assessed for either lectin or mAb binding.

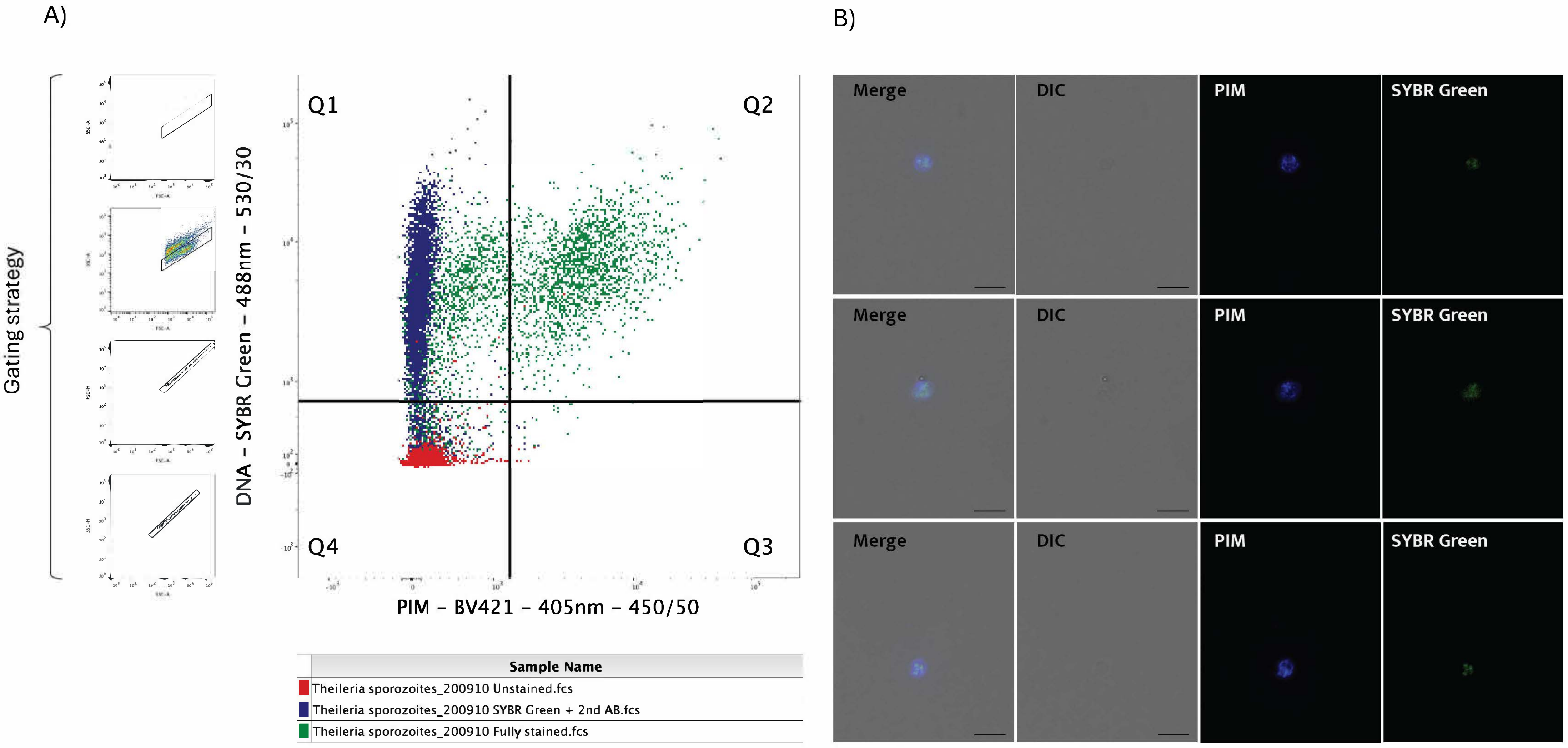

Supplementary Figure 2 Detection of enriched TpM schizonts via flow cytometry

TpM schizonts enriched from 1 x 108 infected cells were stained for the abundant polymorphic immunodominant molecule (PIM) on the parasite surface and schizont DNA. A) Identification of all events in comparison to filtered sheath fluid alone on a BD FACSAria™ Fusion cell sorter was followed by two doubled discriminations (gating strategy). Events with a fluorescent signal from both PIM and DNA staining (Q2) were sorted for analysis via fluorescence microscopy. The gating for such events was performed conservatively to minimise the analysis and sorting of potential host cell contaminants. Control samples included unstained parasites (red) and schizonts stained with DNA stain and secondary antibody alone (blue). B) Microscopic analysis of sorted events with the 100X oil lens of a Nikon Ti2 microscope showed round areas of about 5 μm with several green fluorescing foci from SYBR Green staining and a violet surface fluorescence from anti-PIM staining.

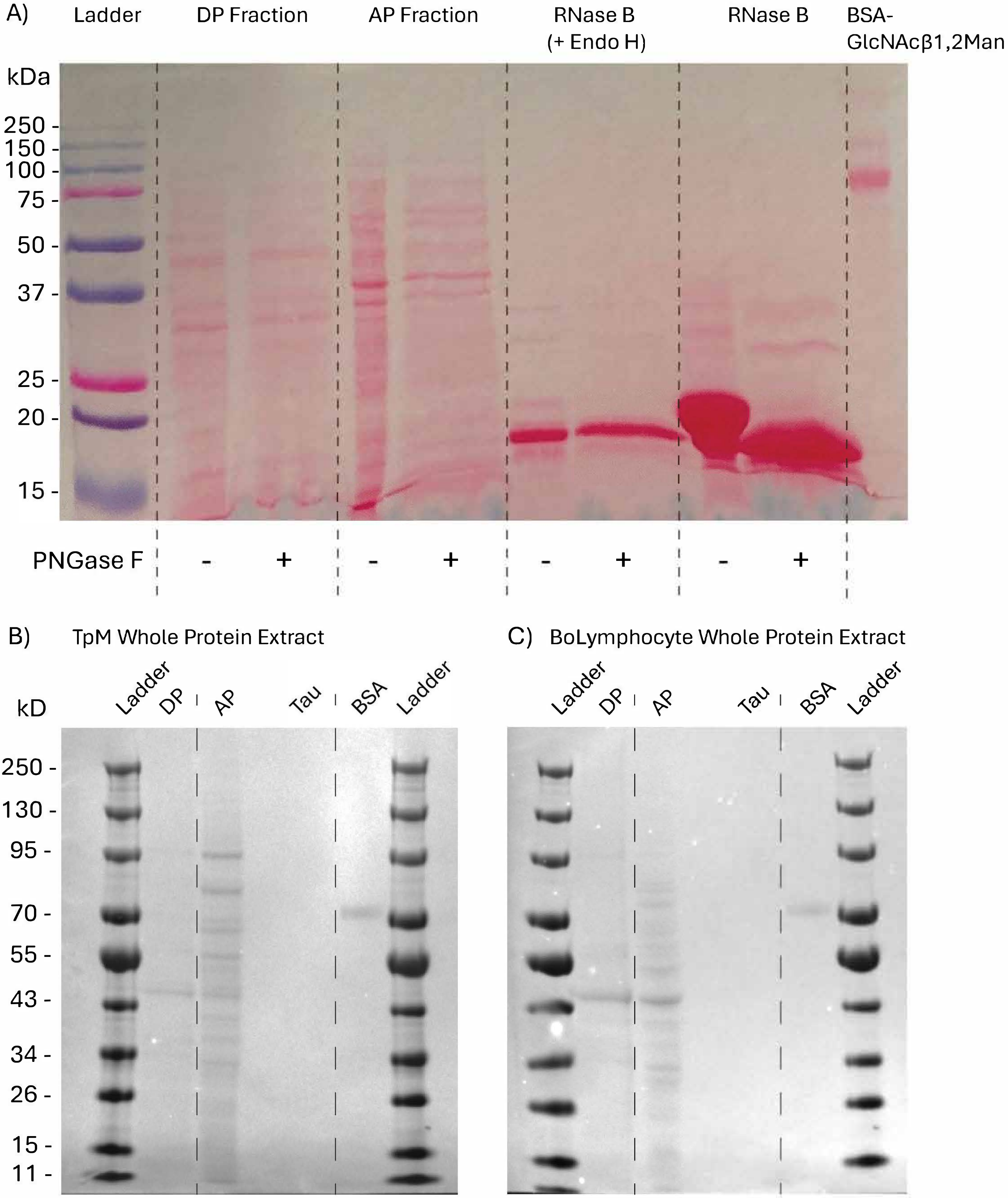

Supplementary Figure 3 Loading controls for Western Blot analyses of protein extract

A) Ponceau staining of the Western Blot membrane shown in Figure 2C confirmed approximately equal loading of untreated and de-N-glycosylated samples for subsequent probing of the membrane with WGA. Bovine ribonuclease (RNase) B with an unmodified (no Endo H treatment) or truncated N-glycoform (post Endo H treatment) was used to confirm de-N-glycosylation of samples via PNGase F and WGA binding specificity, respectively. BSA-GlcNAcβ1,2Man served as negative control. Ponceau staining of the Western Blot membranes shown in Figure 3B-C were imaged in grey scale on an iBright1500 and confirmed approximately equal loading of DP and AP protein fractions from B) enriched TpM schizonts and C) uninfected bovine lymphocytes. Recombinant human Tau-441 and BSA served as positive and negative control, respectively. Tau-441 ran between 80 and 100 kDa due to its glycosylation which led to its very faint ponceau staining.

A)

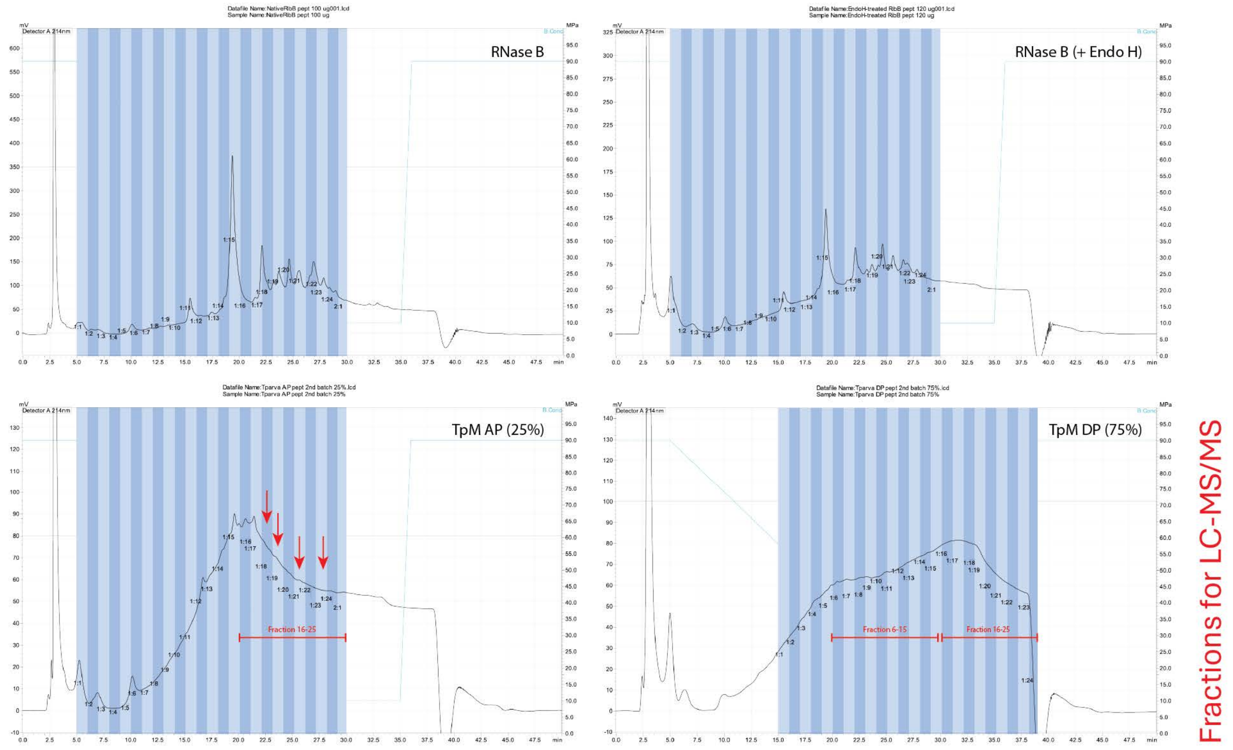

B)

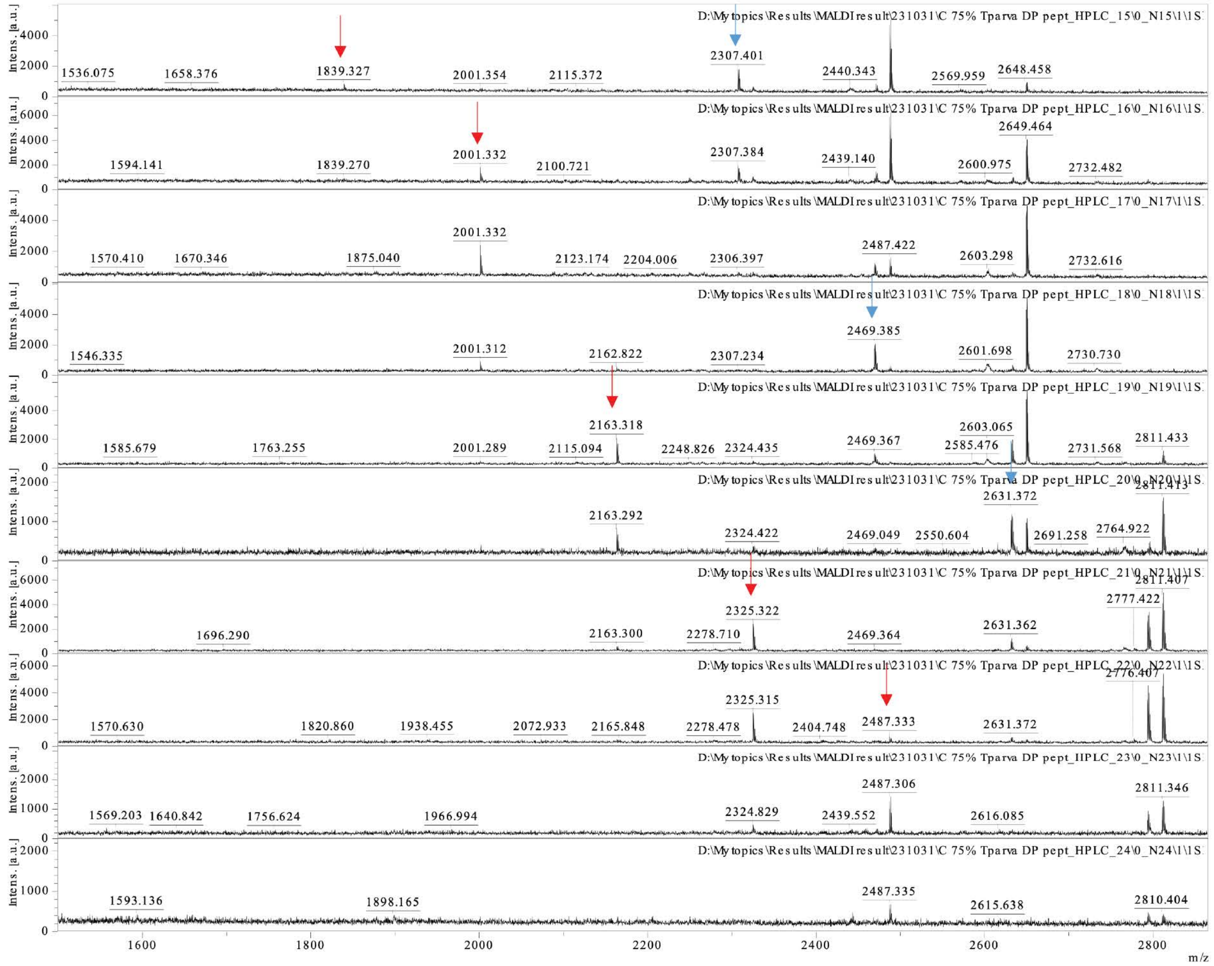

Supplementary Figure 4 HILIC-HPLC and MALDI-TOF MS analysis of TpM schizont peptide fractions for subsequent LC-MS/MS

A) HILIC-HPLC chromatograms of TpM schizont-derived AP and DP tryptic peptides as well as of control samples RNase B and endoglycosidase (Endo) H-treated RNase B. B) MALDI-TOF MS spectra of glycopeptides from the HPLC-enriched TpM schizont DP fraction, displaying peptides carrying hexose oligomers (Δ=162). Red and blue arrows indicate two peptide series.

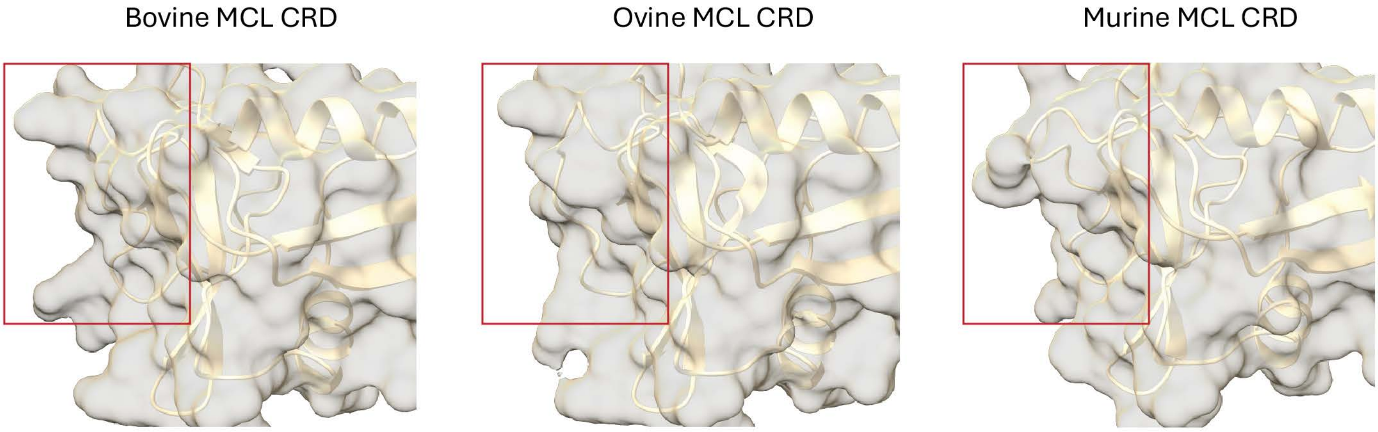

Supplementary Figure 5 Comparison of the ligand binding site of the CRD of bovine, ovine and murine MCL

3D protein structures of the CRD from bovine, ovine (AA82-213) and murine (AA83-218) MCL were generated with AlphaFold3 Server and assessed using ChimeraX-1.9. The region that would contain the canonical sugar-binding site of C-type carbohydrate-recognition domains of other C-type lectin receptors (red rectangle) was identified as described in (32).
