# Supplementary figures and images for: Investigation of carbohydrate-based molecules of Theileria parva parasites
Source: Front Vet Sci. 2026 Jun 8;13:1816563. doi: 10.3389/fvets.2026.1816563 (PMC13283990; doi:10.3389/fvets.2026.1816563)

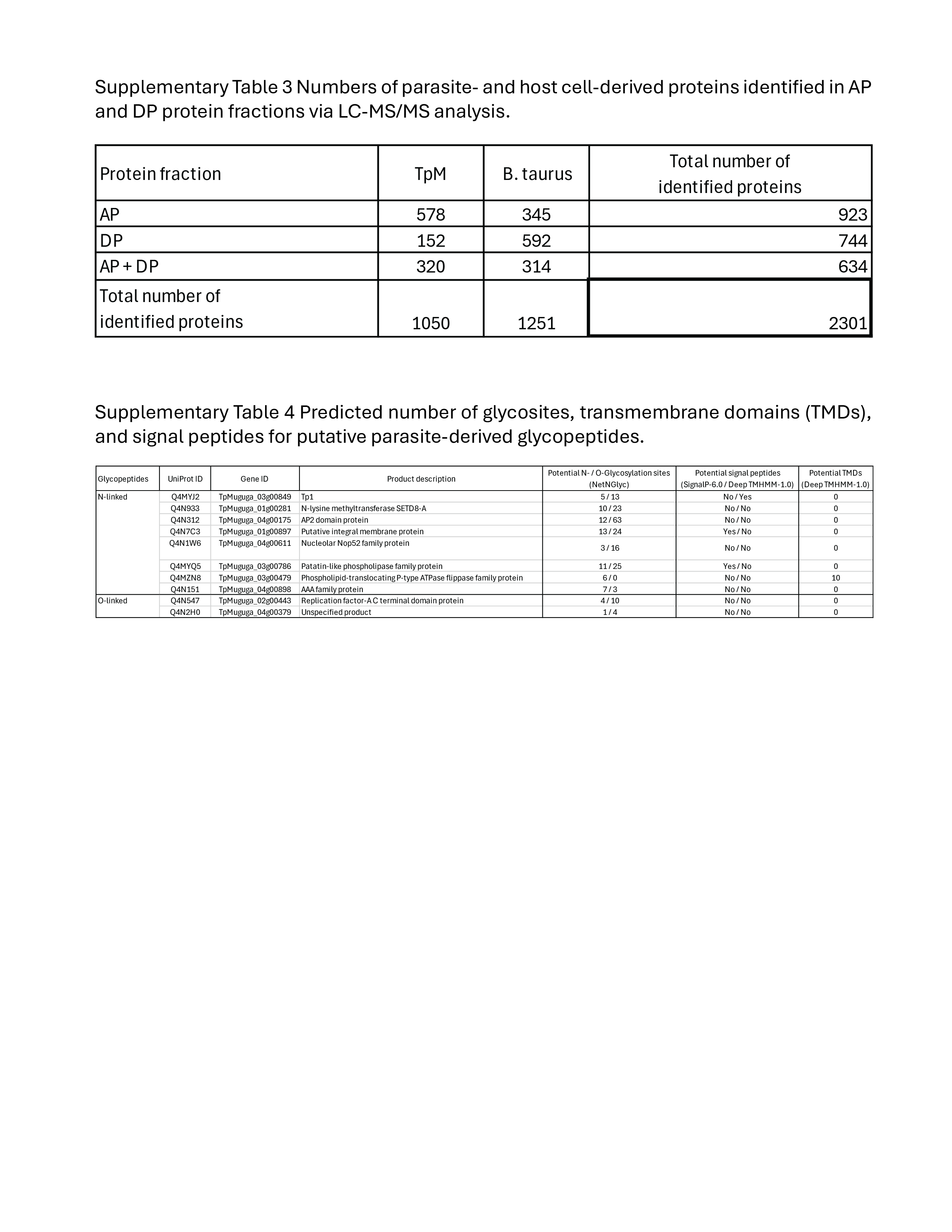

Supplement: SUPPLEMENTARY FIGURE 1 — Gating strategies for flow cytometric screenings of enriched TpM schizonts for glycosylated surface molecules. TpM schizonts were enriched from infected cells and stained with (A) different CLR-hFc-fusion proteins, (B) WGA with or without prior incubation with different concentrations of GlcNAc or (C) an anti-O-GlcNAc-specific mAb. Flow cytometric identification of all events compared to filtered sheath fluid alone was followed by two doublet discriminations (gating strategy). Single events with a fluorescent signal from (A + C) DNA staining or (B) anti-PIM staining were assessed for either lectin or mAb binding. [file Supplementary_file_1.zip › Supplementary_Material/Supplementary Tables 3 and 4.TIF]
